# Cryptic genetic variation of expression quantitative trait locus architecture revealed by genetic perturbation in *Caenorhabditis elegans*

**DOI:** 10.1093/g3journal/jkad050

**Published:** 2023-03-02

**Authors:** Marijke H van Wijk, Joost A G Riksen, Mark Elvin, Gino B Poulin, Muhammad I Maulana, Jan E Kammenga, Basten L Snoek, Mark G Sterken

**Affiliations:** Laboratory of Nematology, Wageningen University & Research, Droevendaalsesteeg 1, 6708 PB Wageningen, The Netherlands; Laboratory of Nematology, Wageningen University & Research, Droevendaalsesteeg 1, 6708 PB Wageningen, The Netherlands; Peak Proteins, Birchwood House, Larkwood Way, Macclesfield, Cheshire, SK10 2XR, UK; Division of Molecular and Cellular Function, School of Biological Sciences, Faculty of Biology, Medicine and Health, University of Manchester, Manchester Academic Health Science Centre, Manchester, Oxford Road, M13 9PL, UK; Laboratory of Nematology, Wageningen University & Research, Droevendaalsesteeg 1, 6708 PB Wageningen, The Netherlands; Laboratory of Nematology, Wageningen University & Research, Droevendaalsesteeg 1, 6708 PB Wageningen, The Netherlands; Theoretical Biology and Bioinformatics, Utrecht University, 3584 CH Utrecht, The Netherlands; Laboratory of Nematology, Wageningen University & Research, Droevendaalsesteeg 1, 6708 PB Wageningen, The Netherlands

**Keywords:** *C. elegans*, cryptic genetic variation, eQTL, genetic perturbation

## Abstract

Genetic perturbation in different genetic backgrounds can cause a range of phenotypes within a species. These phenotypic differences can be the result of the interaction between the genetic background and the perturbation. Previously, we reported that perturbation of *gld-1*, an important player in the developmental control of *Caenorhabditis elegans*, released cryptic genetic variation (CGV) affecting fitness in different genetic backgrounds. Here, we investigated the change in transcriptional architecture. We found 414 genes with a *cis-*expression quantitative trait locus (eQTL) and 991 genes with a *trans-*eQTL that were specifically found in the *gld-1* RNAi treatment. In total, we detected 16 eQTL hotspots, of which 7 were only found in the *gld-1* RNAi treatment. Enrichment analysis of those 7 hotspots showed that the regulated genes were associated with neurons and the pharynx. Furthermore, we found evidence of accelerated transcriptional aging in the *gld-1* RNAi–treated nematodes. Overall, our results illustrate that studying CGV leads to the discovery of hidden polymorphic regulators.

## Introduction

Cryptic genetic variation (CGV) is genetic variation that generates little or no phenotypic variation in a population under normal conditions. The effect of this hidden genetic variation can be unlocked by environmental or genetic perturbation (Gibson and Dworkin 2004). A classic example of CGV due to mutational perturbation was reported for the fruit fly *Drosophila melanogaster*. The effect of mutations in genes involved in wing development on wing shape was tested in different fly genetic backgrounds. It appeared that the effect of the mutation differed strongly across the different genetic backgrounds (Dworkin and Gibson 2006). Hidden genetic variation is widespread across species and affects numerous complex traits including disease and disorder (Merlo and Boyle 2003; Chen *et al*. 2016; Riordan and Nadeau 2017; Niepoth and Bendesky 2020). Investigating the interaction between a gene perturbation in different genetic backgrounds facilitates the detection of hidden polymorphic regulators of complex traits.

The model nematode species *Caenorhabditis elegans* allows for studying CGV due to its genetic tractability and the possibility to combine forward and reverse genetic perturbation tools with populations of recombinant inbred lines (RILs) (Li *et al*. 2006; Gutteling *et al*. 2007; Viñuela *et al*. 2010). Studies that focus on CGV in *C. elegans* have used quantitative trait locus (QTL) analysis, with the aim of detecting polymorphic loci associated with trait variance between RILs (Evans *et al*. 2021). Many QTL studies in *C. elegans* have focused on CGV due to environmental perturbations (Rodriguez *et al*. 2012; Balla *et al*. 2015; Zdraljevic *et al*. 2017; Evans *et al*. 2018; Zamanian *et al*. 2018; Snoek *et al*. 2019). But quantitative genetics studies investigating the phenotypic response due to genetic perturbation are less well studied. These studies have the potential to reveal “modifier loci” affecting the mutant phenotype (Milloz *et al*. 2008; Duveau and Felix 2012). Genetic perturbation in QTL studies can be accomplished by introgressing a mutation in a RIL population, inserting a mutation via CRISPR/Cas9, or by exposing the RILs to an RNAi treatment to create a loss or reduction of function phenotype. Elvin *et al.* (2011) used RNAi to knock down specific genes in an N2xCB4856 RIL population and subsequently measured the fitness effects. QTL close to *ppw-1* (a PAZ/PIWI protein) were found for several different genetic perturbations. The gene *ppw-1* is known to be involved in germline sensitivity to RNAi (Tijsterman *et al*. 2002; Elvin *et al*. 2011); however, although *ppw-1* plays a role, it is not the only genetic factor determining the phenotypic response (Pollard and Rockman 2013). Moreover, the penetrance of the RNAi treatment has been shown to be a complex interplay between target gene and genetic background (Paaby *et al*. 2015; Vu *et al*. 2015).

Gene expression QTL (eQTL) reflect the genetic architecture of complex traits (Cookson *et al*. 2009). eQTL are polymorphic loci associated with gene expression variation and can be mapped either locally (in *cis*) or distantly (in *trans*). *Cis*-eQTL are eQTL where DNA variation within the affected gene causes expression variation, whereas genes of *trans*-eQTL are distantly regulated by, for example, a transcription factor. Within this paper, we will refer to *cis-*eQTL as locally mapped eQTL and to *trans-*eQTL as distantly mapped eQTL. Many t*rans*-eQTL can map to the same genomic region forming clusters called *trans*-bands (eQTL hotspots). It is these *trans-*eQTL and *trans-*bands that are affected the most by environmental and genetic perturbations (Viñuela *et al*. 2012; Sterken *et al*. 2017). In the context of cryptic genetic variation, eQTL studies aim to reveal the hidden transcriptional network. Environmental and genetic perturbations have revealed eQTL patterns in *C. elegans* (Viñuela *et al*. 2010; Snoek *et al*. 2017; Sterken *et al*. 2017). Viñuela *et al*. (2010) found that variation in gene expression increases with age, and that *trans-*acting eQTL are relatively more abundant than *cis-*acting eQTL in older nematodes compared to younger nematodes. Furthermore, genetic perturbations by introgression of the gain-of-function mutation of *let-60* in an N2xCB4856 RIL population showed mostly effects on *trans*-eQTL (Sterken *et al*. 2017). Taken together, these studies paint a picture of the transitory and reactive nature of *trans*-eQTL to perturbation.

Here, we studied CGV that is released upon perturbation of *gld-1*. The gene *gld-1* encodes for an RNA-binding protein involved in the regulation of meiotic entry and oocyte development in *C. elegans* and is an important target of the *glp-1*/NOTCH pathway. This signaling pathway is highly conserved across species and has been implicated with key cell fate decisions such as proliferation, differentiation, and cellular reprogramming (Artavanis-Tsakonas *et al*. 1999; Jarriault *et al*. 2008; Liu *et al*. 2010; Greenwald and Kovall 2013). Differences in the genetic background can influence an animal's physiological response to *gld-1* perturbation, as exposing N2xCB4856 RILs to *gld-1* RNAi releases CGV that affects fitness traits (Elvin *et al*. 2011; Vu *et al*. 2015).

We extended the study of Elvin *et al*. (2011) by investigating the transcriptional architecture after *gld-1* perturbation by exposing an N2xCB4856 RIL population to *gld-1* RNAi. The penetration of the *gld-1* RNAi treatment can differ per strain, as germline sensitivity for RNAi was found to be different across genotypes and the responsible underlying loci are segregated in this RIL population (Tijsterman *et al*. 2002; Elvin *et al*. 2011; Pollard and Rockman 2013; Paaby *et al*. 2015). Therefore, the gene expression changes and the mapped eQTL will not only gain insight into the molecular mechanisms, pathways, and modifiers associated with *gld-1*, it will also reveal CGV associated with *gld-1* RNAi. We found that *gld-1* perturbation had a major impact on global gene expression and that transcriptional aging was accelerated in *gld-1* RNAi–treated animals compared to animals treated with an empty vector. eQTL analysis showed distinct *trans-*eQTL patterns between the *gld-1* RNAi–treated and the empty vector–treated animals.

## Materials and methods

### Strains used and culturing

The *C. elegans* strains used in this study were the wild-type strains Bristol N2 and CB4856 and 48 RILs derived from a cross between N2 and CB4856 (genotypes are described in Li *et al*. 2006). The RILs used were genetically characterized by SNP markers and low-coverage sequencing (Li *et al*. 2006; Thompson *et al*. 2015). The strains were kept on nematode growth medium (NGM) with *E. coli* OP50 as a food source at 12°C (Sulston and Hodgkin 1988).

### RNAi exposure

The RNAi bacteria clones were kindly provided by Julie Ahringer. The control (empty vector) used in this experiment was L4440. Part of the *gld-1* sequence (T23G11.3, from WormBase), excluding both UTRs, was PCR amplified and inserted in the L4440 backbone, hereby obtaining the *gld-1* vector (Kamath *et al*. 2003).

To ensure consistency between the current and previous experiments, RNAi exposure was performed following the earlier described method and exposed in one single batch (Fraser et al. 2000; Kamath *et al*. 2001, 2003; Elvin *et al*. 2011). In short, gravid adults were bleached, and eggs were allowed to hatch in M9 buffer on a rocking platform at 20 RPM resulting in synchronized L1 larvae. Approximately 200 L1 larvae were deposited on 9-cm RNAi plates seeded with 600 μl of an overnight culture of empty vector (control) or *gld-1* RNAi bacteria and left to grow at 20°C for 72 h (Elvin *et al*. 2011). Gravid adults were harvested in M9 and bleached. Embryos were washed in M9, placed onto freshly seeded RNAi plates, and incubated at 20°C for 47 h. L4 stage nematodes were harvested with M9 buffer, centrifuged, and stored at −80°C until further use. The parental strains were treated with the *gld-1* RNAi and empty vector twice, 39 RILs were exposed to the empty vector, and 46 RILs were exposed to the *gld-1* RNAi treatment (Supplementary Table 1).

### RNA isolation, cDNA synthesis, and labeling

The mRNA was isolated with the RNEasy Micro kit from Qiagen (Hilden, Germany) according to the manufacturer's protocol (purification of total RNA from animal and human tissues) with a modified lysing procedure according to Volkers *et al*. (2013). Labeling of samples, hybridization of the arrays, and feature extraction were done following the manufacturer's instructions in the “Two-Color Microarray-Based Gene Expression Analysis—Low Input Quick Amp Labeling” manual (Agilent Technologies, Santa Clara, CA, USA).

### Data analysis

Data was analyzed using R (version 3.6.1 Windows x64) (Team 2019), and for processing the data, the ggplot2, dplyr, and limma packages were used (Ritchie *et al*., 2015; Wickham, 2016; Wickham *et al*., 2020). Scripts that were used for this project are available online (https://git.wur.nl/published_papers/vanwijk_etal_2020_).

### Microarray hybridization and normalization

The input of total RNA was 200 ng for each sample on *C. elegans* (V2) Gene Expression Microarray 4X44K slides. The microarrays were scanned using an Agilent High Resolution C Scanner using the settings as recommended in the abovementioned manual. Data was extracted with the Agilent Feature Extraction Software version 10.5, following manufacturer's guidelines. For normalization, the limma package for the “R” environment (version 2.13.1 x64) was used (Ritchie *et al*. 2015). No background correction of the RNA array data was performed as recommended (Zahurak *et al*. 2007). For within-array normalization of the RNA array data, the Loess method was used, and for between-array normalization, the quantile method was used (Smyth and Speed 2003). The obtained log_2_-normalized intensities (single channel data) were used for further analysis. Data is accessible at ArrayExpress under E-MTAB-9742.

### Treatment response

The transcriptional responses to the RNAi treatments were determined by explaining the gene expression over the treatments with a linear model,


$$y_{i}\;∼\;T+e_{i}$$

where *y* is the log_2_-normalized intensity as measured by the microarray spot *i* (*i* = 1, 2, …, 45,220) and *T* is treatment (either empty vector or *gld-1* RNAi treatment). Genotype was not taken into account in this analysis. The Bonferroni method in *P_adj_* (*P* < 0.05) was applied over the significances to correct for multiple testing (Benjamini and Yekutieli 2001).

The gene expression data of the RILs was used in a principal component analysis (PCA). Therefore, the data was transformed to a log_2_ ratio with the mean of the 2 treatments using the formula:


$$R_{i.j}=\mathrm{lo}g_{2}\left(\frac{y_{i.j}}{{\bar{y}}_{i}}\right)$$

where *R* is the log_2_ relative expression of spot *i* (*i* = 1, 2, …, 45,220) in strain *j* (RIL) and *y* is the intensity of spot *i* in strain *j*. The function *prcomp* with scale = TRUE, which normalizes the input data, was used to perform the PCA.

### Developmental variation between the RILs

The relative age of the samples was estimated by their transcription profiles. A genetic ruler consisting of 2,195 spots with a significant [log_10_(*P*) > 6 and effect size > 0.1 per hour] linearly up- or downregulation during the time period from 41.5 to 72 h was used, based on a previously published method and data (Snoek *et al*. 2014). The raw data used for this analysis can be obtained from E-MTAB-7019. The expression data from our samples, the same spots that were used to generate the genetic ruler, were compared to the genetic ruler to estimate the transcriptional age of our samples. The relative age was calculated by transforming the predicted ages (in hours and derived from the genetic ruler) into the standard normal distribution.

### eQTL mapping and threshold determination

Expression quantitative trait loci mapping was done using a linear model on the log_2_-normalized intensities. For the empty vector and *gld-1* RNAi treatment, a single-marker model was used,


$$y_{i.j}\;∼\;x_{j}\;+\;e_{j}$$

where *y* is the log_2_-normalized intensity as measured by the microarray of spot *i* (i = 1, 2, …, 45,220) of RIL *j*. This is explained over the genotype (either CB4856 or N2) on marker location *x* (*x* = 1, 2, …, 729) of RIL *j*.

Genome-wide thresholds were determined by permutations per spot per treatment. In this permutation, the log_2_-normalized intensities were randomly distributed over the strains. The randomized data set was then used in eQTL mapping to determine the false discovery rate (FDR) (Benjamini and Yekutieli 2001). This was repeated for 10 randomized data sets. To calculate the genome-wide thresholds, the following formula was used:


$$\frac{\mathrm{FDS}}{\mathrm{RDS}}\leq \frac{m_{0}}{m}q\cdot \log\;(m)$$

where FDS (false discoveries) was the outcome of the permutations and RDS (real discoveries) the outcome of the eQTL mapping at *q* = 0.05. The value of *m* (number of hypotheses tested) was set at 45,220, representing the number of spots on the microarray. The *m*_0_ (number of true hypotheses tested) was 45,220 RDS. In this way, the genome-wide thresholds of −log_10_(*P*) > 3.9 and −log_10_(*P*) > 3.7 were determined for the empty vector and the *gld-1* treatments, respectively. The threshold of −log_10_(*P*) > 3.9 was used for further analysis for both treatments.

### Statistical power analysis

The statistical power for eQTL mapping in the RIL populations was determined in a power analysis (Sterken *et al*. 2017). Per marker, random noise was introduced based on a standard normal distribution with sigma = 1 and mu = 0. For each marker, eQTL were simulated that explained 20, 25, 30, …, 80% of the variance on top of the random introduced noise. Based on the permutation, the threshold of −log_10_(*P*) > 3.9 was used to determine the number of correctly detected QTL, the number of false positives, and the number of undetected QTL. This power analysis also included the precision of the QTL location and the effect size estimation.

### eQTL analysis

After eQTL mapping, distinction was made between *cis*- and *trans*-eQTL based on the position of the gene and the eQTL peak. An eQTL was assigned as *cis* if the peak of the eQTL was within 1 Mb of the gene; other eQTL were assigned *trans* (Snoek *et al*. 2017).

The amount of variance that each eQTL explained was calculated by ANOVA, by analysis of the gene expression over the marker underlying the eQTL peak. This analysis was conducted per peak if spots had multiple eQTL. The eQTL were compared between the empty vector and *gld-1* RNAi treatment by filtering the spots with a similar eQTL type (*cis* or *trans*). *Trans-*eQTL were overlapping when eQTL had overlapping confidence intervals.

To define the *trans-*bands in this experiment, the genome was divided in 203 bins of 0.5 Mb, and *trans*-eQTL were counted per bin. Based on the Poisson distribution (Rockman *et al*. 2010; Snoek *et al*. 2017), it was calculated how many *trans*-eQTL a bin should contain in order to represent an overrepresentation (*P* < 0.0001) and thus contain a *trans*-band.

All eQTL data and results are available on WormQTL2 (Snoek *et al*. 2020).

### Functional enrichment analysis

Gene group enrichment analysis was performed by filtering genes of interest through several databases provided by WormBase.org: the WS220 gene class annotations, the WS256 GO-annotation, anatomy terms, phenotypes, RNAi phenotypes, developmental stage expression, and disease-related genes (Harris *et al*. 2014). Modencode.org provided the MODENCODE release 32 transcription factor binding sites that were mapped to transcription start site (Gerstein *et al*. 2010; Niu *et al*. 2011; Tepper *et al*. 2013). Genome.jp/keg/ provided the KEGG pathway release 65.0 (Ogata *et al*. 1999). Enrichments were selected if the size of category was *n* > 3 and the overlap between genes of interest and enrichment group was *n* > 2. Selected enrichments were tested using a hypergeometric test. Here, we corrected the *P*-values for multiple testing using the Bonferroni correction.

### Comparing eQTL datasets

For determining if there were similar *trans*-bands between this study and Viñuela *et al.* (2010), *trans*-bands were called with the aforementioned method (Viñuela *et al*. 2010; Snoek *et al*. 2017). First, overlapping *trans*-bands were determined by filtering for common *trans*-bands between the 2 data sets. Secondly, it was investigated if the two overlapping *trans*-bands contained identical genes with a *trans-*eQTL. Finally, a hypergeometric test was conducted (*phyper*) to determine if the overlap of identical genes within a *trans*-band or the overlap of *cis*- and/or *trans*-eQTL was greater than we could expect by chance.

## Results

### *gld-1* RNAi has a major impact on gene expression and transcriptional aging

**Fig. 1.**
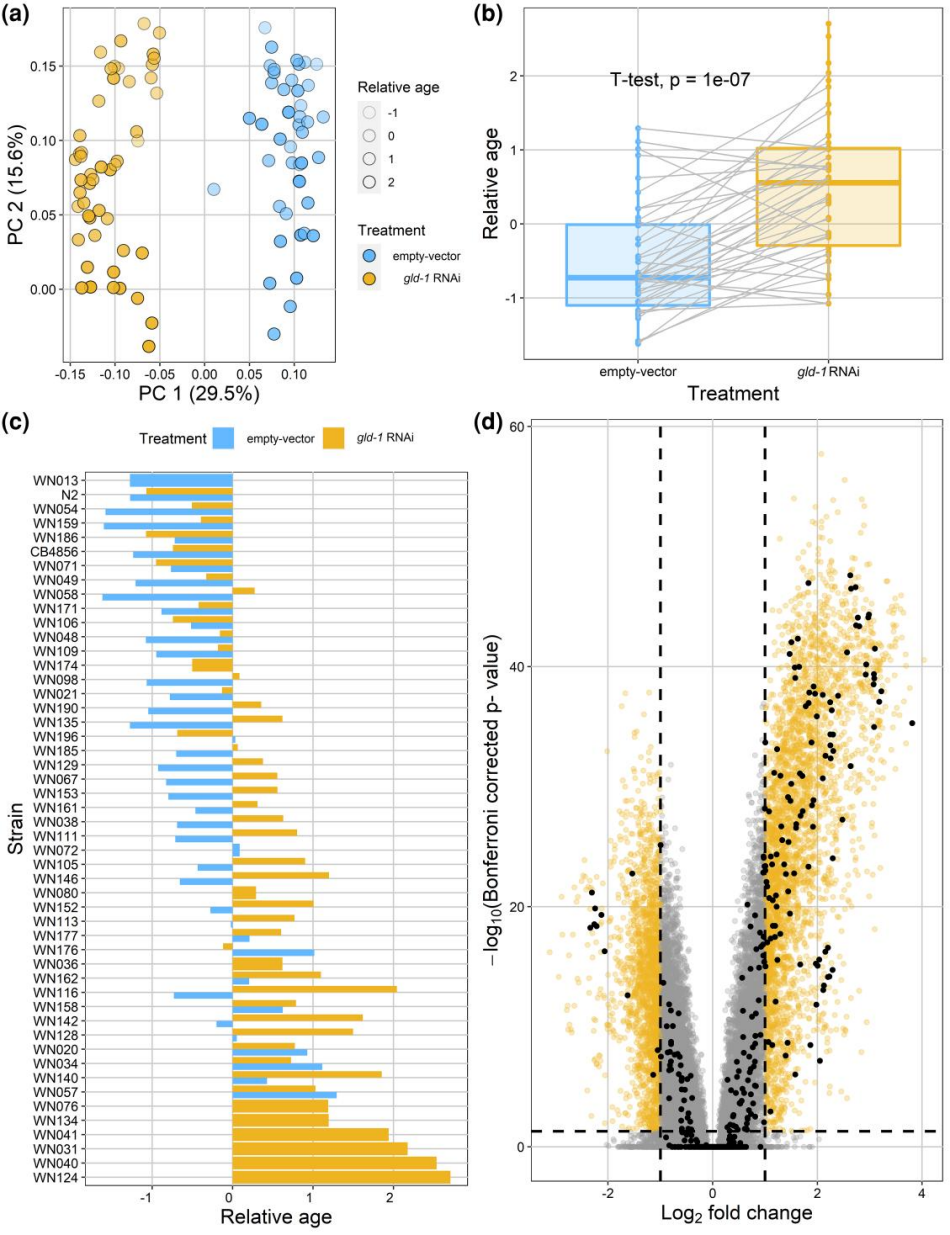
*gld-1* RNAi has a major effect on global gene expression. a) Most of the of the variance in gene expression of the complete data set can be explained by differences in RNAi treatment (29.5%) as measured by principal component analysis (PCA). PC2 is linked to transcriptional age differences. Every dot represents a strain that had been subjected to either the *gld-1* RNAi or the empty vector treatment. b) Strains that underwent the *gld-1* RNAi treatment are significantly transcriptionally older compared to animals that had been exposed to the empty vector (1-tailed *t*-test *P* = 1 × 10^−7^). c) The relative age is influenced by genotype and treatment. d) Higher expression of 1,927 genes and lower expression of 1,056 genes in the *gld-1* RNAi treatment compared to the empty vector treatment (Bonferroni, *P_adj_* < 0.05 and log_2_ fold change > 2). Also, 58 out of 131 genes with a known interaction with *gld-1*/GLD-1 were differentially expressed (Bonferroni, *P_adj_* < 0.05 and log_2_ fold change > 2) which is more than we can expect by chance (hypergeometric test, *P* < 3.2 × 10^–15^). Every dot represents a microarray spot, and some genes are represented by multiple spots. Yellow spots represent the significantly regulated spots. Black spots represent the spots representing genes that have a known interaction with *gld-1*/GLD-1.

We measured gene expression in a set of RILs derived from N2 and CB4856 that were exposed to 2 treatments (empty vector and *gld-1* RNAi). To determine how gene expression variance could be explained by the 2 treatments, we conducted a principal component analysis (PCA). The first axis of the PCA captured the variance that separated the 2 treatments and accounted for 29.5% of the variance in this experiment, indicating that the *gld-1* RNAi treatment caused significant changes in global gene expression when compared to the empty vector treatment (Fig. 1a). The second axis captured 15.6% of the variance. From previous experiments, we knew that RILs have differences in developmental timing (Snoek *et al*. 2019) which can be reflected by changes in gene expression; therefore, it is likely that PC2 was related to differences in transcriptional age (Viñuela *et al*. 2012; Snoek *et al*. 2014) (Fig. 1a).

To further investigate the potential transcriptional age differences, we used previously published data on gene expression over the L4 stage to estimate the relative transcriptional age of the RILs (Snoek *et al*. 2014). Here, we used genes that are linearly regulated over the L4 stage and used them to infer the relative age of the nematodes in our RIL population using gene expression data. As expected, PC2 was indeed linked to differences in transcriptional age, although all animals were harvested 47 h after bleaching and subsequent RNAi exposure (Fig. 1a). Strains that were exposed to the *gld-1* RNAi treatment were, on average, transcriptionally older than empty vector–treated strains (1-tailed *t*-test, *P* < 1 × 10^−6^), indicating that accelerated aging is induced by *gld-1* RNAi treatment (Fig. 1b).

Comparing the strains, treatments, and relative age showed the complex interplay between genotype and RNAi treatment on transcriptional age (Fig. 1c). It is notable that the differences in transcriptional age between the empty vector and the *gld-1* RNAi treatment are relatively small between the parents N2 and CB4856, whereas much larger differences in transcriptional age can be observed in the RIL progeny. If this increased transcriptional age was solely caused by natural variation in *ppw-1*, an important gene in germline RNAi sensitivity, then, we would expect much larger differences in relative transcriptional age between the empty vector and *gld-1* RNAi treatments between N2 and CB4856. Furthermore, RILs showed a pattern of transgressive behavior for relative transcriptional age in either the empty vector or the *gld-1* RNAi treatment and for the difference between relative transcriptional age between the empty vector and *gld-1* RNAi treatment. This indicates that multiple alleles are segregating in this population that influence transcriptional aging after *gld-1* RNAi exposure (Fig. 1c).

### Perturbation effects are primarily due to *gld-1* RNAi, not RNAi itself

Observations on the level of gene expression are in line with the observations on population growth (Elvin *et al*. 2011). Namely, *gld-1* RNAi exposed populations grew faster compared to populations that were fed with an empty vector (Supplementary Fig. 1a). Population growth was determined by adding *C. elegans* to a 96-well plate with *E. coli*, containing the appropriate RNAi vector, and to subsequently measure the OD_600_ at different time points. Here, a high relative OD_600_ meant less food had been consumed and translated to a small nematode population, whereas a low relative OD_600_ meant that more food had been consumed and translated to a larger nematode population. Moreover, if the differences in fitness between the RILs were solely the result of RNAi penetration variation instead of *gld-1* perturbation, we would expect all target genes to behave the same in a particular RIL. However, we clearly see that each RNAi construct has a genotype-dependent effect on fitness. This means that natural variation in fitness after *gld-1* RNAi perturbation is primarily caused by *gld-1* perturbation (Supplementary Fig. 1a).

The transcriptional age is correlated with population growth effects. Because there were 39 identical RILs used between our study and the study of Elvin *et al.* (2011), we reanalyzed their data and compared the inferred transcriptional age of our samples to the OD_600_ of their samples. We observed that the largest differences in OD_600_ could be observed between 96 and 144 h (Supplementary Fig. 1a). Next, we correlated the relative age of the samples from our study with the normalized OD_600_ and found that the relative transcriptional age negatively correlated with the OD_600_ (Pearson correlation 96 h: *P* < 0.005, 120 h: *P* < 0.01, 144 h: *P* < 0.05) (Supplementary Fig. 1b). Therefore, we conclude that the transcriptionally older RILs in our study were also growing faster in the study of Elvin *et al.* (2011).

Next, we wanted to explore the extent and variety of differentially expressed genes between *gld-1* RNAi and empty vector–treated animals. We found that 1,927 genes were higher expressed in the *gld-1* RNAi treatment compared to the empty vector and 1,056 genes were lower expressed in the *gld-1* treatment compared to the empty vector (Bonferroni, *P_adj_* < 0.05 and log_2_ fold change > 2) (Fig. 1d) (Supplementary Table 2). Enrichment of genes that were higher expressed in the empty vector condition were mainly involved in neuronal anatomy and oxidation processes, whereas genes that were higher expressed in the *gld-1* RNAi–treated animals were mainly involved in embryo development and reproduction (Supplementary Table 3). To see whether the RNAi pathway was differentially regulated between the 2 treatments, we obtained a list of genes known to be involved in the RNAi pathway (e.g. *alg-1*, *dcr-1*, and *eri-1*) from WormBase. We found that 5 out of 27 genes contained transcripts that were significantly higher expressed in the *gld-1* RNAi treatment compared to the empty vector treatment (Bonferroni, *P_adj_* < 0.05 and log_2_ fold change > 2) (Supplementary Table 4). Therefore, we must conclude that gene expression differences between the 2 treatments were the result of *gld-1* perturbation and the effect of RNAi itself.

Because *gld-1* is known to be an important translational regulator, we investigated for the transcriptional response of known *gld-1* interaction targets. We previously observed a positive correlation between mRNA and protein abundance in N2 and CB4856 (Kamkina *et al*. 2016). Therefore, we hypothesized that if there would be a change in GLD-1 abundance, there would be a change in protein concentration of translational GLD-1 targets which would influence GLD-1 target gene mRNAs. We obtained a list of genes that have a genetic, physical, regulatory, and/or predicted interaction with *gld-1* from WormBase. Of these 209 known interactions, we were able to detect the transcription levels of 131 unique genes. In total, we found 58 genes that were differentially expressed as a result of the *gld-1* RNAi treatment, which is more than we can expect by chance alone (Fig. 1d) (Bonferroni, *P_adj_* < 0.05 and log_2_ fold change > 2 and hypergeometric test, *P* < 3.2 × 10^−15^) (Supplementary Table 5).

All in all, the clear separation between the *gld-1* RNAi–treated animals and the empty vector animals in the PCA (Fig. 1a), the difference in transcriptional age between the 2 treatments (Fig. 1b and c), the significant correlation between the transcriptional ages of the samples in this study and the population fitness of the samples in Elvin *et al.* (2011) (Supplementary Fig. 1, a and b), the large number of differentially expressed genes between the 2 treatments (Fig. 1d), and the overrepresentation of differentially expressed genes that are known to be interacting with *gld-1*/GLD1 provide evidence of *gld-1* perturbation.

### Differences in eQTL architecture between the empty vector and *gld-1* RNAi treatment

**Fig. 2.**
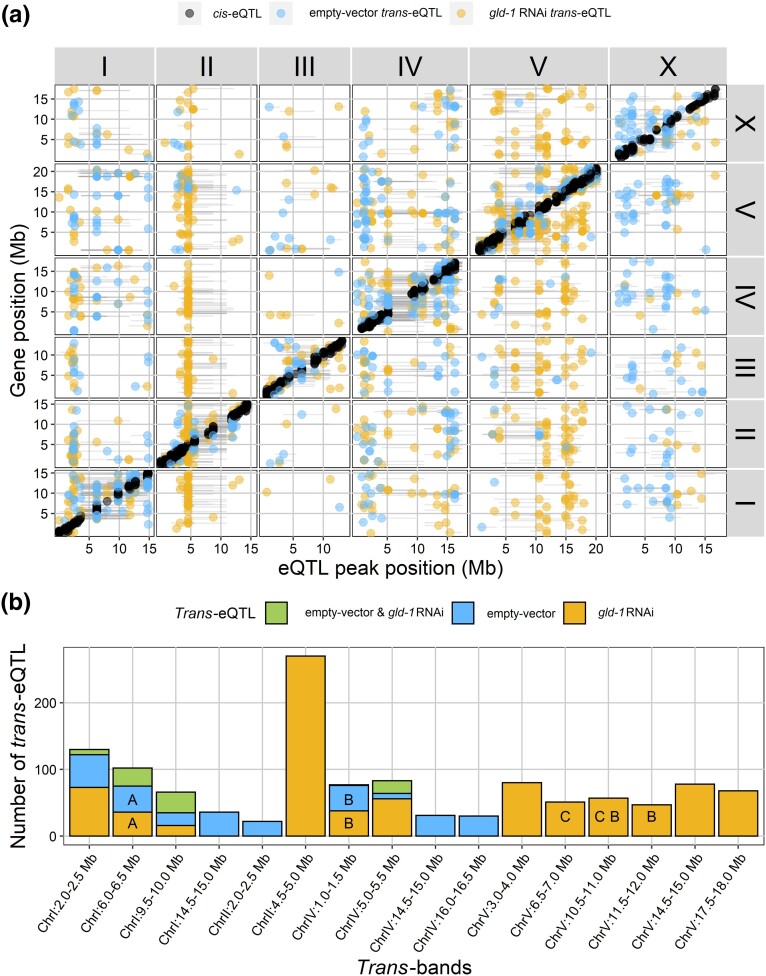
eQTL mapped in this experiment. a) The black dots represent the spots with a *cis-*eQTL and the blue/gold dots represent the spots with a *trans*-eQTL detected in the empty vector or *gld-1* RNAi treatment, respectively. The *y*-axis represents the genomic location of the differentially expressed spots; the *x*-axis represents the genomic location of the mapped eQTL. The gray horizontal lines represent the confidence interval of the eQTL (based on a 1.5 drop in −log_10_(*P*). b) *Trans-*bands that are found in this study can be specific for the empty vector treatment or the *gld-1* RNAi treatment or can colocate and share some eQTL (Poisson distribution, *P* < 0.0001). Numbers of *trans-*eQTL are represented in spots, not genes. *Trans-*bands that were found in Viñuela *et al.* (2010) that share more eQTL with eQTL in our *trans*-bands at that specific *trans-*band interval than can be expected by chance are marked with a letter (A, juvenile; B, adult; C, old) (hypergeometric test, *P* < 0.05).

To investigate the effect of the genetic variation on gene expression after *gld-1* perturbation, we mapped eQTL. We used a single-marker model for the eQTL mapping and compared the identified eQTL (FDR ≤ 0.05) between the empty vector and *gld-1* RNAi treatment. Statistical power analyses showed that the populations represented in the empty vector and the *gld-1* RNAi treatment had the power to detect 64.7 and 78.9% of the eQTL that explain at least 35% of the variance, respectively (Supplementary Table 6). In total, we found 1,358 genes with an eQTL in the empty vector treatment and 1,949 genes with an eQTL in the *gld-1* RNAi treatment (Fig. 2a) (Supplementary Table 7). This difference in the number of eQTL can partly be explained by the difference in statistical power between the data sets. *Trans-*eQTL often explain less variance compared to *cis-*eQTL and are therefore more likely to be missed when calling eQTL.

To compare eQTL between the 2 treatments, we defined shared *cis-*eQTL as genes with an eQTL and classified as *cis*-eQTL (eQTL that map within 1.0 Mb of the physical location of the gene), whereas shared *trans-*eQTL were genes with eQTL that were classified as *trans-*eQTL (eQTL that do not map within 1.0 Mb of the physical location of the gene) and had overlapping confidence intervals. We found 531 genes with a *cis*-eQTL that overlapped between the empty vector and the *gld-1* RNAi treatment and 240 and 414 genes with a *cis*-eQTL that were only observed in the empty vector and *gld-1* RNAi treatment, respectively (Table 1; Fig. 2a). Enrichment of *cis*-eQTL of each treatment individually yielded gene classes, *bath*, *math*, and *pals*, which were also found in previous eQTL studies in *C. elegans* (Volkers *et al*. 2013; Snoek *et al*. 2017; Sterken *et al*. 2017). Furthermore, we found 76 genes with a *trans*-eQTL that overlapped between the empty vector and the *gld-1* RNAi treatment; 566 and 991 genes with a *trans*-eQTL were only found in the empty vector or *gld-1* RNAi treatment, respectively (Table 1; Fig. 2a).

**Table 1.** Number of genes with an eQTL per RNAi treatment. Numbers of spots on the microarray with an eQTL that represent the number of genes with an eQTL are depicted in bold.

	*gld-1*	Empty vector
Specific *cis-*eQTL	414 genes, **513 spots**	240 genes, **298 spots**
Overlap *cis-*eQTL	531 genes, **779 spots**	
Total *cis-*eQTL	900 genes, **1,292 spots**	742 genes, **1,077 spots**
Specific *trans-*eQTL	991 genes, **1,533 spots**	556 genes, **891 spots**
Overlap *trans-*eQTL	76 genes, **234 spots**	
Total *trans-*eQTL	1,049 genes, **1,767 spots**	616 genes, **1,125 spots**
Total eQTL	1,949 genes, **3,059 spots**	1,358 genes, **2,202 spots**

Because *trans-*eQTL typically form hotspots of loci that regulate the transcription of many genes, we determined the location of hotspots in our data. *Trans-*eQTL hotspots were identified by comparing the number of *trans-*eQTL in a specific bin to the average number of eQTL mapped in the experiment (Poisson distribution, *P* < 0.0001). In total, we found 9 *trans*-bands for the empty vector treatment and 12 *trans*-bands for the *gld-1* RNAi treatment. Most *trans*-bands harbored *trans*-eQTL that were specific for either the *gld-1* RNAi treatment or the empty vector condition. However, *trans-*eQTL hotspots on chromosomes I and IV contained *trans*-eQTL that were treatment specific but also included *trans-*eQTL that were shared between treatments (Fig. 2b). These shared eQTL are most likely to be an underrepresentation because of differences in statistical power between the data sets. However, this might also suggest that regulatory loci underlying those *trans-*eQTL hotspots change transcription specificity after *gld-1* perturbation.

To elucidate the underlying biology of the different *trans-*bands, we performed an enrichment analysis on the genes that mapped to each *trans-*band. The genes that mapped to the *trans-*eQTL hotspots in the *gld-1 trans*-bands revealed enrichment for protein (de)phosphorylation (ChrII:4.5–5.0 Mb); transcription factors (ChrIV:1.0–1.5 Mb); endopeptidase activity (ChrV:6.5–7.0 Mb); nerve system, hormone activity, and phenotypes associated with movement (ChrV:14.5–15.0 Mb); and unfolded protein response (ChrV:17.5–18.0 Mb).

Perturbation using *gld-1* RNAi not only revealed the genetic architecture of *gld-1* perturbation-specific effects; it also may have revealed RNAi-specific processes since RNAi penetration is genotype dependent (Elvin *et al*. 2011; Paaby *et al*. 2015; Vu *et al*. 2015). Elvin *et al.* (2011) discovered that natural variation in *ppw-1* explained the part of the RNAi penetration variation between N2 and CB4856. Here, we did not find an eQTL for *ppw-1* or a *trans-*band that mapped to the *ppw-1* genomic location (ChrI:4.2 Mb). Furthermore, we did not find differences in RNAi pathway–specific eQTL between the 2 treatments, nor did we observe *trans-*bands that mapped to the genomic location of important RNAi genes that contain predicted high impact variation between N2 and CB4856 (Supplementary Table 4). Therefore, we can conclude that RNAi penetration differences between genetic backgrounds were unlikely to have a major impact on the eQTL mapping.

### The eQTL architecture upon *gld-1* RNAi indicates increased aging

Because we had found an accelerated transcriptional aging effect upon *gld-1* RNAi perturbation, we were curious to see if we could find age-related eQTL patterns in our data. Therefore, we used the eQTL dataset of Viñuela *et al*. (2010) where eQTL patterns in *C. elegans* at 3 different ages were investigated: juveniles (40 h), adults (96 h), and old nematodes (214 h) (Viñuela *et al*. 2010). When we compared our eQTL to the juvenile eQTL data, we found that one *trans*-band (ChrI:6.0–6.5 Mb) had more overlapping eQTL between the empty vector and juveniles and the *gld-1* RNAi and juveniles than could be expected by chance (hypergeometric test, *P* = 1.5 × 10^−11^ and 3.8 × 10^−8^, respectively) (Fig. 2b). This indicates that the *trans-*band ChrI:6.0–6.5 Mb between the 3 conditions is the same and might be regulated by the same regulator. When we compared our empty vector *trans*-bands to *trans-*bands from the adult and old nematode life stages, we only found 1 *trans*-band (ChrIV:1.0–1.5 Mb) with more overlapping eQTL than we could expect by chance (hypergeometric test, *P* < 7.0 × 10^−5^). Strikingly, when we compared the *gld-1* RNAi treatment *trans*-bands to the *trans-*bands from the adult and old nematode life stages, we found 5 *trans-*bands (adult, ChrIV:1.0–1.5 Mb, ChrV:10.5–11.0 Mb, and ChrV:11.5–12.0 Mb; old nematodes, ChrV:6.5–7.0 Mb and ChrV:10.5–11.0 Mb) with more overlapping eQTL than we could expect by chance (hypergeometric test, *P* = 2.7 × 10^−3^, 2.7 × 10^−2^, 1.8 × 10^−11^, 4.3 × 10^−3^, and 4.6 × 10^−4^, respectively). This relatively large number of overlapping *trans-*bands between the *gld-1* RNAi eQTL and the adult and old nematode eQTL of Viñuela *et al.* (2010) points towards increased aging processes upon *gld-1* perturbation.

## Discussion

### Genes linked to *gld-1* are affected by CGV

In our study, we observed a clear effect in gene expression caused by the *gld-1* RNAi treatment (in the PCA, in the DEG analysis, transcriptional age). Elvin *et al.* (2011) and Vu *et al.* (2015) observed a clear phenotypic effect when animals were exposed to *gld-1* RNAi compared to control animals (Elvin *et al*. 2011; Vu *et al.* 2015). We expected to find lower levels of *gld-1* in the *gld-1* RNAi–treated animals compared to the empty vector–treated animals. However, we observed a transcriptional increase of *gld-1*, indicating a feedback mechanism for obtaining adequate GLD-1, like in Brenner and Schedl (2016). There they found that *gld-1[gld-1(q485)/+]* animals showed similar phenotypic features compared to full *gld-1* knockouts, indicating that a lower gene dose of *gld-1* has an effect on the animals. However, GLD-1 levels in adult *gld-1[gld-1(q485)/+]* nematodes were normal, suggesting a feedback mechanism between *gld-1* and GLD-1 (Brenner and Schedl 2016). Investigating the precise interaction between *gld-1* and GLD-1 is beyond the scope of this paper but may be an interesting topic for future research.

The genetic perturbation caused by *gld-1* RNAi will have varied between genotypes since germline sensitivity depends on the genetic background (Tijsterman *et al*. 2002; Pollard and Rockman 2013; Paaby *et al*. 2015; Vu *et al*. 2015). Much of the phenotypic variance between N2 and CB4856 after RNAi treatments can be attributed to allelic differences in *ppw-1*; CB4856 harbors a natural null allele, making CB4856 more resistant to the effects of germline active RNAi (Tijsterman *et al*. 2002; Elvin *et al*. 2011; Pollard and Rockman 2013; Vu *et al*. 2015). However, both Vu *et al.* (2015) and Elvin *et al.* (2011) showed that the phenotypic variation between N2 and CB4856 after *gld-1* RNAi can only partially be explained by natural variation in *ppw-1*. Furthermore, we did not observe an eQTL for *ppw-1* nor did we find a *trans-*band located at the *ppw-1* locus, indicating that allelic variation in *ppw-1* did not have a large effect on the perturbated transcriptional architecture. Taken together, we conclude that any phenotypic effects in this RIL panel were the result of a combination of *gld-1* RNAi penetration and genetic background effects.

Next to an abundance of differentially regulated genes, we found many different *trans-*eQTL and *trans*-bands due to *gld-1* RNAi, confirming that *trans-*eQTL are affected by genetic perturbations (Sterken *et al*. 2017). However, it should be noted that differences in eQTL observed in this study are influenced by multiple factors, i.e. statistical power differences between the 2 experimental conditions, developmental effects, natural variation in RNAi specificity, mishybridization on the microarray due to polymorphisms, and the *gld-1* perturbation. Off-target effects of the used *gld-1* RNAi construct could have altered gene expression of unintended target genes (Jackson *et al*. 2003). However, we found no differences in eQTL of genes that are involved in the RNAi pathway between the 2 treatments nor did we find *trans-*bands at genomic locations harboring polymorphic RNAi genes. This suggests that genetic variation did not strongly affect gene expression of RNAi, therefore indicating that the RNAi itself did not have a large influence on genetic architecture.

### Perturbation with *gld-1* RNAi affects transcriptional aging

Development plays an important role in the detection of eQTL. (e)QTL patterns can be influenced by developmental speed differences within a RIL population and have been observed before (Snoek *et al*. 2019; Snoek *et al*. 2021). Furthermore, eQTL patterns change with age (Viñuela *et al*. 2010). In this study, we found that *gld-1* RNAi–treated animals were transcriptionally older than the empty vector–treated animals. Moreover, when we compared eQTL patterns between our data and Viñuela *et al*. (2010), we observed that *gld-1* RNAi–treated animals shared more *trans-*bands with adult and old nematodes than the empty vector animals share with adult and old nematodes. This is interesting since the *glp-1*/NOTCH pathway has been implicated in the development of age-related diseases in human and thus might play an important role in aging in general (Balistreri *et al*. 2016). A possible explanation for the enhanced transcriptional age of the *gld-1* RNAi–treated nematodes is the connection between the NOTCH pathway and insulin/IGF-1 signaling. The downregulation of *gld-1* could influence *glp-1* expression and translation, which in turn might act on insulin/IGF-1 signaling genes (Marin and Evans 2003; Schaffitzel and Hertweck 2006). Upregulation of the insulin/IGF-1 pathway promotes reproductive development, thereby aging the nematodes (Schaffitzel and Hertweck 2006). Further investigations into the interplay between genetic background, altered transcriptional age, and *gld-1* perturbation might be an interesting research topic for the future.

### Linking eQTL architecture to previously observed fitness responses

This study started as an extension of Elvin *et al.* (2011) where they exposed an N2xCB4856 RIL panel to *gld-1* RNAi and mapped the fitness responses. Comparison of *trans*-bands mapped in this research with QTL peaks associated with fitness found by Elvin *et al.* (2011) resulted in the overlap of 2 regions: ChrI:6:0–6:5 Mb and ChrIV:16.0–16.5 Mb. The QTL on chromosome IV in Elvin *et al.* (2011) was mapped in the empty vector and in the *gld-1* RNAi treatment, but in our study, we only found a *trans*-band at that location for the empty vector treatment. The overlapping *trans-*band on chromosome I in our study is present in the empty vector treatment and the *gld-1* RNAi treatment, whereas Elvin *et al.* (2011) only mapped a QTL in the *gld-1* RNAi treatment and attributed it to *ppw-1*. The gene *ppw-1* has been implicated with germline sensitivity to RNAi and is located on chromosome I at 4.2 Mb. However, no *trans-*band was found in our research on that locus and neither did we find an eQTL for *ppw-1*. This suggests that natural variation in gene expression of *ppw-1* does not explain differences in germline sensitivity to RNAi, which is in line with the natural variation segregated in the RIL panel, which includes the CB4856 null allele (Tijsterman *et al*. 2002; Elvin *et al*. 2011; Pollard and Rockman 2013; Vu *et al*. 2015). Furthermore, the *ppw-1* null allele does not influence germline sensitivity to RNAi via the regulation of many transcripts.

### Conclusion

Here, we present the transcriptional response and the contribution of CGV on eQTL between empty vector– and *gld-1* RNAi–exposed animals. The mapped eQTL might explain part of the underlying genetics of the differences in fitness observed in Elvin *et al.* (2011) and could potentially help to elucidate differences in other phenotypic traits between N2 and CB4856 related to *gld-1* perturbation. Furthermore, we provided evidence that *gld-1* perturbation is causing accelerated transcriptional aging in *C. elegans*. In conclusion, we demonstrate that genetic perturbation causes genotype-dependent shifts in transcriptional architecture that most likely affect phenotypic differences. Therefore, we not only suggest to take natural variation into account for studying the complex genetic architecture of the *glp-1*/NOTCH pathway but also when studying the effects of other important genetic perturbations.

## Supplementary Material

jkad050_Supplementary_Data

## Data Availability

Scripts that were used for this project are available online (https://git.wur.nl/published_papers/vanwijk_etal_2020_). Transcriptome data were deposited at ArrayExpress under E-MTAB-9742. eQTL results are publicly accessible on WormQTL2. Supplemental material available at G3 online.
