# Supplementary material for: Cryptic genetic variation of expression quantitative trait locus architecture revealed by genetic perturbation in Caenorhabditis elegans
Source: G3 (Bethesda). 2023 Mar 2;13(5):jkad050. doi: 10.1093/g3journal/jkad050 (PMC10151397; doi:10.1093/g3journal/jkad050)
Supplement: jkad050_Supplementary_Data [file jkad050_supplementary_data.zip › Supplemental_Material_Legends_G3-2022-404027.docx]

**Table and figure legends supplements**

*Table S1*

An overview of the strains used in this study. The numbers represent the number of replicates.

*Table S2*

Comparison of gene transcription levels between the empty-vector and *gld-1* RNAi treatment using a linear model. In this linear model genotype was not considered.

*Table S3*

Enrichment analysis of the differentially expressed genes. Enrichment was done on two groups: genes that were significantly higher expressed in the *gld-1* RNAi treatment and genes that were significantly higher expressed in the empty-vector treatment (both Bonferroni, p.adj < 0.05 & log2fold change > 2).

*Table S4*

An overview of genes involved in the RNAi pathway. The transcription levels of those genes were compared between the empty-vector and *gld-1* RNAi treatment (Bonferroni, p.adj < 0.05).

*Table S5*

Differentially expressed transcripts upon *gld-1* RNAi treatment with a known interaction with *gld-1* (obtained from WormBase) (Bonferroni, p.adj < 0.05, log_2_fold change > 2).

*Table S6*

Power analysis to determine statistical power of the RIL populations in this experiment.

*Table S7*

An overview of the eQTL mapped in this study (-log10(p) > 3.9).

**Figure S1: Comparison of fitness data generated in Elvin et al (2011) with our data. A:** The fitness response to different RNAi constructs is target gene dependent. The lower the OD600, the lower the concentration of *E. coli* in the growth medium. **B:** The normalized OD600 that we obtained from Elvin et al (2011), significantly correlates with the inferred transcriptional age of our samples at 96, 120, and 144 hours (Pearson correlation, p < 0.05). Indicating that relative transcriptionally older RILs from our study have faster growing populations in Elvin et al (2011).
