# Supplementary figures and images for: Cryptic genetic variation of expression quantitative trait locus architecture revealed by genetic perturbation in Caenorhabditis elegans
Source: G3 (Bethesda). 2023 Mar 2;13(5):jkad050. doi: 10.1093/g3journal/jkad050 (PMC10151397; doi:10.1093/g3journal/jkad050)

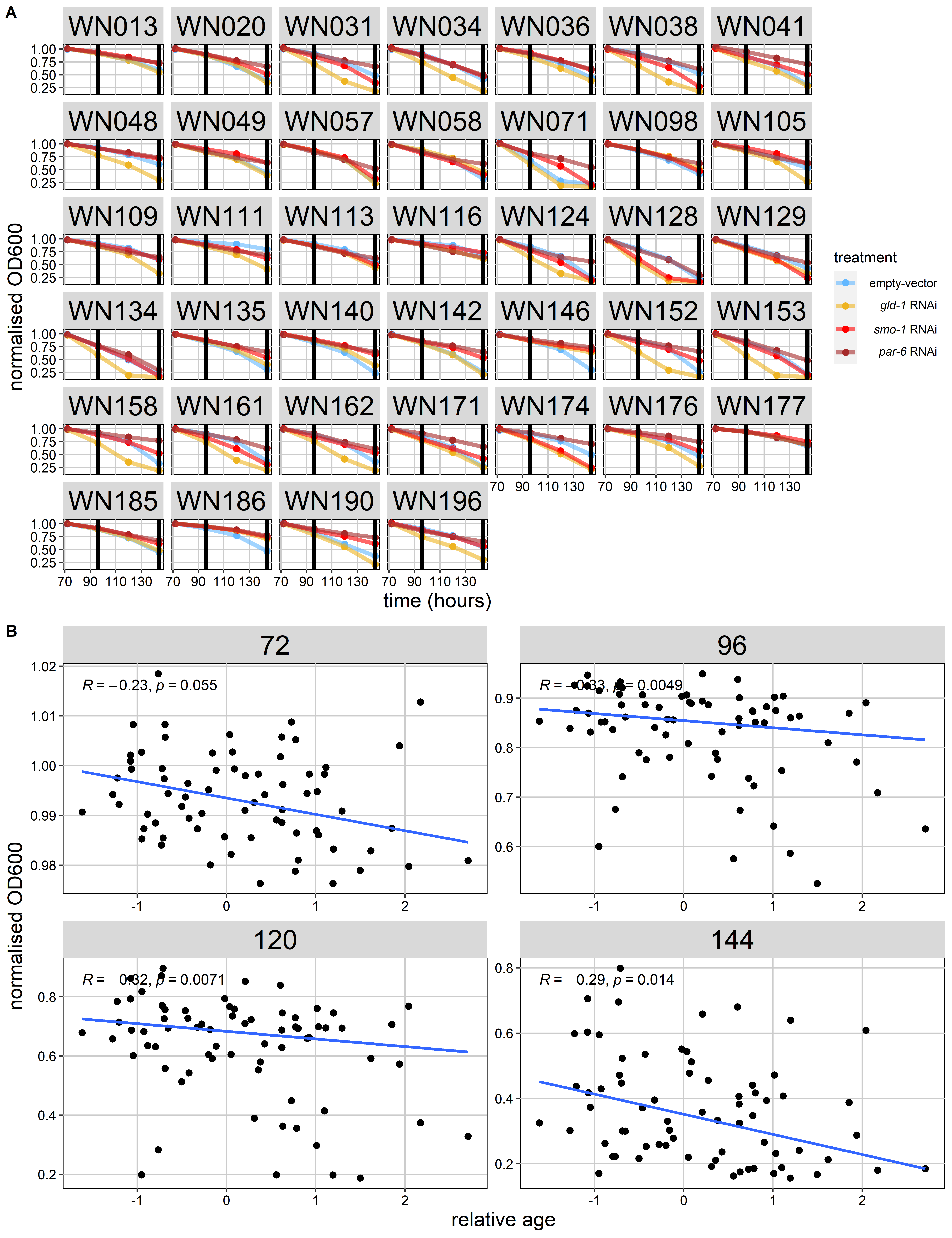

Supplement: jkad050_Supplementary_Data [file jkad050_supplementary_data.zip › Supplemental_Figure_1_G3-2022-404027.png]
